# Better healthcare can reduce the risk of COVID-19 in-hospital post-partum maternal death: evidence from Brazil

**DOI:** 10.1093/ije/dyac157

**Published:** 2022-08-10

**Authors:** Char Leung, Li Su, Ana Cristina Simões e Silva

**Affiliations:** School of Clinical Medicine, University of Cambridge, Cambridge, UK; School of Clinical Medicine, University of Cambridge, Cambridge, UK; Faculty of Medicine, Universidade Federal de Minas Gerais (UFMG), Belo Horizonte, Brazil

**Keywords:** COVID-19, Brazil, SARS-CoV-2, post-partum, healthcare

## Abstract

**Objective:**

COVID-19 in post-partum women is commonly overlooked. The present study assessed whether puerperium is an independent risk factor of COVID-19 related in-hospital maternal death and whether fatality is preventable in the Brazilian context.

**Methods:**

We retrospectively studied the clinical data of post-partum/pregnant patients hospitalized with COVID-19 gathered from a national database that registered severe acute respiratory syndromes (SIVEP-Gripe) in Brazil. Logistic regressions were used to examine the associations of in-hospital mortality with obstetric status and with the type of public healthcare provider, adjusting for socio-demographic, epidemiologic, clinical and healthcare-related measures.

**Results:**

As of 30 November 2021, 1943 (21%) post-partum and 7446 (79%) pregnant patients of age between 15 and 45 years with COVID-19 that had reached the clinical endpoint (death or discharge) were eligible for inclusion. Case-fatality rates for the two groups were 19.8% and 9.2%, respectively. After the adjustment for covariates, post-partum patients had almost twice the odds of in-hospital mortality compared with pregnant patients. Patients admitted to private (not-for-profit) hospitals, those that had an obstetric centre or those located in metropolitan areas were less likely to succumb to SARS-CoV-2 infection. Those admitted to the Emergency Care Unit had similar mortality risk to those admitted to other public healthcare providers.

**Conclusion:**

We demonstrated that puerperium was associated with an increased odds of COVID-19-related in-hospital mortality. Only part of the risk can be reduced by quality healthcare such as non-profit private hospitals, those that have an obstetric centre or those located in urban areas.

Key Messages

- COVID-19 in post-partum women is commonly overlooked.
- Post-partum women had almost twice the odds of COVID-19 in-hospital mortality compared with pregnant patients.
- Part of the mortality risk is preventable through quality healthcare such as the presence of an obstetric centre in the hospital, non-profit private healthcare and hospitals in urban areas.

## Introduction

Coronavirus disease 2019 (COVID-19) is caused by the severe acute respiratory syndrome coronavirus 2 (SARS-CoV-2, formerly known as 2019-nCoV). It is one of the seven coronaviruses pathogenic to humans and is one of the three coronaviruses that cause acute respiratory disease syndrome in humans, along with MERS-CoV and SARS-CoV.^1^ As a respiratory disease, symptoms of COVID-19 can vary from mild ones such as cough to dyspnea in severe cases.^2^ Respiratory failure and cytokine release syndrome are common causes of death.^3^

To improve COVID-19-related healthcare and public health policies, research has been done to identify COVID-19 mortality and morbidity risk factors in population groups that are believed to be more prone to infection. Recognizing the suppressed maternal immune system,^4^ a number of studies have investigated the impact of SARS-CoV-2 infection on maternal health.^5–7^ In contrast, health in post-partum women has been overlooked. Observations emerged that post-partum women might be more prone to COVID-19-related mortality or morbidity.^8^ A multicenter study in the USA reported post-partum exacerbations with hypoxia, although no death was reported.^9^ Studies in Brazil on COVID-19 and pregnancy reported that deaths were concentrated in the third trimester or post-partum.^10^^,^^11^ These observations warrant a further investigation to clarify the risk of death in post-partum. Nevertheless, COVID-19-related deaths in post-partum women in Brazil might be attributed to relatively poor and inaccessible healthcare compared with the USA.

Effort has been made to improve the public healthcare in Brazil. The Unified Health System (SUS, Sistema Único de Saúde) is the government-funded body responsible for healthcare policies that aim to increase the healthcare coverage to the Brazilian population. A recent one is the creation of Emergency Care Units (UPAs, Unidade de Pronto Atendimento)^12^ that are open to the public 24 h for pre-hospital care. Because hospitals are conventionally the entry point for healthcare in Brazil,^13^ UPAs are attached to hospitals and aim to support hospitals by dealing with cases requiring the level of care beyond primary care.^14^ UPAs have gained popularity throughout the country since the creation in the state of Rio de Janeiro in 2007. The number of UPAs in Brazil increased by 122% between 2011 and 2016.^15^ However, UPAs do not replace hospital care as severe cases are normally sent for proper hospital care. During the COVID-19 pandemic in Brazil, the UPAs assumed the care of severe patients due to the lack of hospital beds.

Against this background, the present study aimed to assess the impact of puerperium on COVID-19 related in-hospital maternal death, taking into consideration socio-demographic and healthcare-related factors such as healthcare quality and accessibility. We also further assessed whether UPAs contributed to reducing in-hospital mortality risk.

## Method

### Selection of study cohort

The study cohort was cases registered in SIVEP-Gripe, a nationwide database managed by the Brazilian government for the surveillance of severe acute respiratory syndrome (SRAG, Síndrome Respiratória Aguda Grave) related to respiratory viruses. Demographic and medical data of patients with SRAG and admitted to hospitals were recorded in the database. Data were systematically registered in a pre-determined form and verified by the medical practitioner at the point of care. The database has been the primary source of information on COVID-19-related hospital admission and deaths in Brazil, and has been described elsewhere.^7^

Data were gathered from the database on 30 November 2021. Cases that met all the following criteria were eligible for inclusion: (i) polymerase chain reaction (PCR)-positive for SARS-CoV-2, (ii) female aged between 14 and 45 years, (iii) has reached clinical endpoint (recovered, defined as discharged or died), (iv) is pregnant or post-partum (defined as diagnosis with SARS-CoV-2 within 45 days after delivery) at the of diagnosis. Cases failing to meet any of the above inclusion criteria were excluded. Patients who died of causes other than SARS-CoV-2 infection were excluded.

### Study population definition

Included patients were categorized as (i) pregnant or post-partum and (ii) admitted to the UPA or not, for those admitted to only public hospitals.

### Data source/measurement

Data of covariate measurements were also gathered from SIVEP-Gripe. Covariates included socio-demographic factors, epidemiologic characteristics, signs and symptoms, co-morbidities and healthcare-related factors.

Socio-demographic factors included age at diagnosis, obstetric status (first, second, third trimester, post-partum), location by region (Southeast, South, Center West, North and Northeast), ethnicity (Hispanic/African, Caucasian, Asian and indigenous) and current smoker. Epidemiologic characteristics included nosocomial infection, history of exposure to animals, first wave of the pandemic (from the index case in Brazil in February 2020 to end of October 2020 before the P.1 strain emerged causing the second wave^16^), time from symptom onset to admission and time from admission to death/discharge. Signs and symptoms were those at admission and during hospitalization, including asymptomaticity, abnormal chest X-ray (interstitial infiltrate and/or consolidation), anosmia, ageusia, coryza, cough, diarrhoea, dyspnea, fatigue, fever, headache, myalgia, low oxygen saturation (<95% at admission), respiratory discomfort, sore throat, vomit and others. Co-morbidities included chronic diseases (cardiovascular, hematologic, liver, neurological, pulmonary and renal), asthma, cancer, diabetes, Down syndrome, endocrine disease, gestational diabetes, HIV infection, immunocompromised, maternal hypertensive disorder, mental disorder, obesity and respiratory viral infection (viruses that cause the common cold and influenza, confirmed by PCR tests). In addition, a group of variables was constructed to represent the number of co-morbidities in a patient, including ‘no co-morbidities’, ‘one co-morbidity’, ‘two co-morbidities’ and ‘three or more co-morbidities’. Healthcare-related factors included ICU (intensive care unit) admission, ventilation, use of antiviral against influenza, vaccination against influenza, vaccination against SARS-CoV-2, whether the hospital was located in the Metropolitan Region (Regiões Metropolitanas, legally defined in Brazil), whether there was an obstetric centre in the hospital, type of healthcare provider (public, private, not-for-profit private) and whether the patient was admitted to a UPA if using public healthcare. However, ICU admission and ventilation were omitted from the multivariable regression described in the subsequent section.

Obstetric centres were defined by the Observatory of Hospital Policy and Management (OPGH, Observatório de Política e Gestão Hospitalar) that had a pre-delivery room (sala de pré-parto), normal delivery room (sala de parto normal), curettage room (sala de curetagem) and/or operation room (sala de cirurgia). Public healthcare providers were further categorized as UPA or non-UPA.

Complete data were not available for all variables. For any missing data on signs, symptoms (except for abnormal chest X-ray) or co-morbidities, the clinical condition was assumed to be absent, following the approach in previous studies with the same database.^7^^,^^17^ Missing data of abnormal chest X-ray were excluded from the relevant analysis because imaging was not performed. Cases with ethnicity missing were also excluded from the corresponding analysis.

Our study only included patients who reached clinical endpoints (death or discharge), which means that patients who were still being treated at the hospitals were excluded from this study and their endpoint data were missing at the study cut-off date. We assumed that data were missing completely at random for clinical endpoints, abnormal chest X-ray and ethnicity.

### Definition of outcome measures and comparison groups

The primary hypothesis was whether patients in post-partum, relative to pregnancy, were associated with an increased risk of in-hospital mortality. The secondary hypothesis was whether patients admitted to a UPA were associated with an increased risk of in-hospital mortality compared with patients not admitted to a UPA. For both hypotheses, the relative effect measure for in-hospital mortality was estimated by odds ratio (OR), adjusted for socio-demographic factors, epidemiologic characteristics, signs and symptoms, co-morbidities and healthcare-related factors.

### Statistical analysis

Descriptive statistics were computed and compared between the pregnant and post-partum groups. For continuous variables, *t*-tests or Mann-Whitney tests were used for comparison, depending on the validity of the normality assumption. Fisher’s exact tests were used for dichotomous variables.

For the primary hypothesis, the OR for mortality was estimated using a multivariable logistic regression model. For the secondary hypothesis, only patients admitted to public hospitals were considered and cases were further categorized as UPA or non-UPA. The OR for mortality was calculated using a multivariable logistic regression model.

For both outcomes, the forward stepwise procedure was adopted for covariate selection in all regression models with a *P*-value of 10% as the threshold. The area under the receiver operating characteristic (ROC) curve was used to assess the goodness-of-fit of these models. Variance inflation factors (VIFs) were used to assess collinearity. ICU admission and ventilation were omitted from the selection procedure because they were not accurate measures. There was an insufficient number of ICU beds and ventilators during the pandemic.^18^ The Southeast Region was chosen as the reference group for location variables since it had the best and most accessible healthcare in the country. Hispanic/African was selected as the reference group for ethnicity as they formed the largest ethnic group in Brazil. The variable ‘No co-morbidity’ and public healthcare, the predominant healthcare sector in the country, were chosen as the reference group for variables concerning the number of co-morbidities and the type of healthcare provider, respectively.

Sensitivity analysis was performed to assess the robustness of the result. The specified multivariable logistic regression model was fitted to a subset of the data that consisted of severe cases only, defined by low oxygen saturation (<95%).^19^ It has been suggested that there might be a lower threshold for hospitalization of pregnant women,^20^ making them more likely to be admitted to the hospital for precautionary reasons.^21^ Therefore, cases included in the sensitivity analysis had similar disease severity and potential selection bias could be assessed.

All data analyses were performed using R Version 4.1.1 and a *P*-value of <0.05 was deemed statistically significant.

## Results

**Figure 1:**
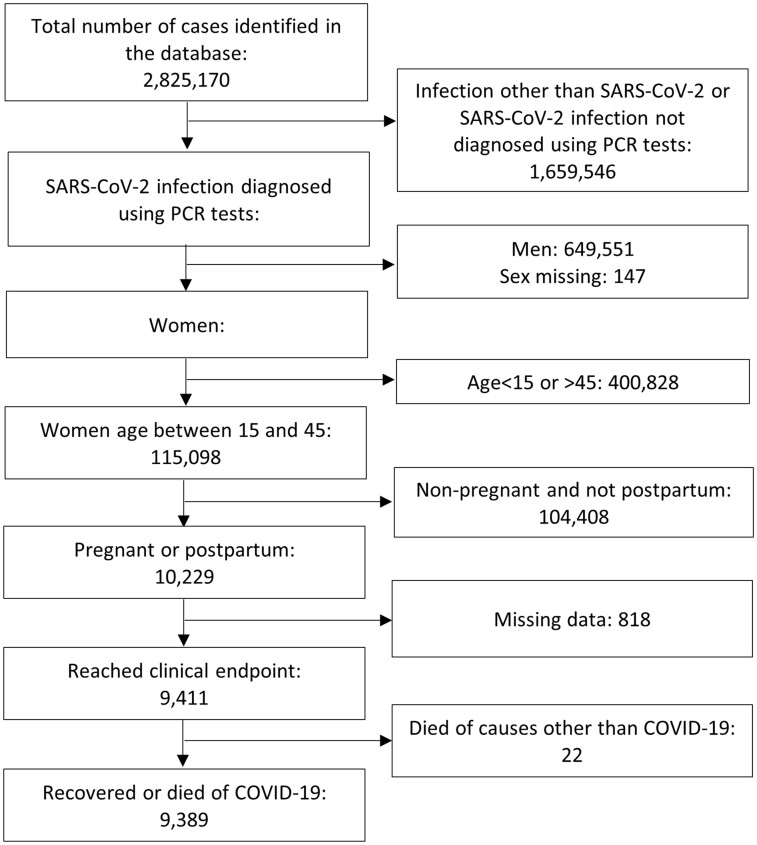
Flow diagram of case selection

A total of 2 825 170 SRAG cases were registered in the database as of 30 November 2021, of which 1 165 624 cases (41%) were PCR-positive for SARS-CoV-2 (Figure 1). These cases comprised 515 926 women (44%), 649 551 men (56%) and 147 cases (<1%) with sex missing. A total of 115 098 (22%) women aged between 15 and 45 years were included. Of these, 10 229 were either pregnant or post-partum (9%) whereas the remaining 104 408 patients were not pregnant and not post-partum (91%). Of those who were pregnant or post-partum, 9411 had reached the clinical endpoint that consisted of 22 patients who died of causes other than COVID-19 (<1%) and hence were removed from the study. The remaining 9389 cases (>99%) were eligible for inclusion. Patients of the included cases were admitted to the hospital between 15 March 2020 and 22 November 2021 with symptom onset dated between 8 March 2020 and 22 November 2021.

Characteristics of the study cohort are shown in Table 1. A total of 1943 (21%) patients were in post-partum whereas 7446 were pregnant (79%). Patients in post-partum had a significantly higher rate of fatality (19.8% vs 9.2%, *P *<* *0.001). Such a difference might be attributed to several factors, as reflected in the difference between the two groups. The post-partum group had a significantly higher median of age (31 vs 30 years, *P *=* *0.017) although the interquartile ranges (IQRs) were similar, at 25–35 years. Furthermore, there was a significantly higher share of post-partum patients than pregnant patients (20.7% vs 15.7%, *P *<* *0.001) in the Northeast Region. Difference in the ethnic composition was also salient with a higher proportion of Hispanic/African (54.8% vs 51.3%, *P *=* *0.015) and a lower proportion of Caucasian (44.1% vs 47.4%, *P *=* *0.016) patients in the post-partum group.

**Table 1:** Characteristics of the study cohort (*n* = 9389)

	Post-partum (*n*)	Pregnant (*n*)	*P*
Socio-demographic			
Maternal age (years) [median (IQR)]	31 (25–35) (1943)	30 (25–35) (7446)	0.017
Dead (%)	19.76 (384/1943)	9.23 (687/7446)	<0.001
Smoker (%)	0.21 (4/1943)	0.48 (36/7446)	0.116
Gestational age (%)			
First trimester	NA	9.33 (695/7446)	NA
Second trimester	NA	28.35 (2111/7446)	NA
Third trimester	NA	62.32 (4640/7446)	NA
Location (%)			
North	6.18 (120/1943)	6.20 (462/7446)	>0.999
Northeast	20.69 (402/1943)	15.74 (1172/7446)	<0.001
Southeast	45.24 (879/1943)	46.29 (3447/7446)	0.414
Center West	11.53 (224/1943)	14.60 (1087/7446)	<0.001
South	16.37 (318/1943)	17.16 (1278/7446)	0.416
Ethnicity (%)			
Caucasian	44.05 (707/1605)	47.41 (2978/6281)	0.016
Asian	0.75 (12/1605)	0.91 (57/6281)	0.653
Hispanic/African	54.77 (879/1605)	51.33 (3224/6281)	0.015
Indigenous	0.44 (7/1605)	0.35 (22/6281)	0.643
Epidemiologic			
Time from symptom onset to admission (days) [median (IQR)]	5 (2–9) (1879)	6 (3–9) (7152)	<0.001
Time from admission to recovery (days) [median (IQR)]	5 (3–11) (1466)	6 (3–10) (6322)	0.001
Time from admission to death (days) [median (IQR)]	13 (6–20) (367)	14 (7–21.25) (672)	0.070
Nosocomial infection (%)	2.78 (54/1943)	0.90 (67/7446)	<0.001
History of exposure to animals (%)	0.57 (11/1943)	0.77 (57/7446)	0.452
First wave (%)	36.18 (703/1943)	33.29 (2479/7446)	0.018
Signs and symptoms (%)			
Asymptomatic	0.77 (15/1943)	0.44 (33/7446)	0.075
Abdominal pain	4.48 (87/1943)	6.33 (471/7446)	0.002
Abnormal chest X-ray	92.96 (383/412)	86.60 (1131/1306)	<0.001
Anosmia	9.57 (186/1943)	14.71 (1095/7446)	<0.001
Ageusia	8.75 (170/1943)	13.13 (978/7446)	<0.001
Coryza	7.57 (147/1943)	9.52 (709/7446)	0.008
Cough	62.74 (1219/1943)	72.68 (5412/7446)	<0.001
Diarrhoea	7.21 (140/1943)	10.13 (754/7446)	<0.001
Dyspnea	56.15 (1091/1943)	59.84 (4456/7446)	0.003
Fatigue	15.65 (304/1943)	20.36 (1516/7446)	<0.001
Fever	49.67 (965/1943)	58.07 (4324/7446)	<0.001
Headache	11.12 (216/1943)	17.26 (1285/7446)	<0.001
Myalgia	7.05 (137/1943)	14.91 (1110/7446)	<0.001
Low oxygen saturation	45.14 (877/1943)	38.42 (2861/7446)	<0.001
Respiratory discomfort	47.25 (918/1943)	44.87 (3341/7446)	0.062
Sore throat	18.73 (364/1943)	21.25 (1582/7446)	0.016
Vomit	5.25 (102/1943)	11.03 (821/7446)	<0.001
Others	35.31 (686/1943)	46.07 (3430/7446)	<0.001
Co-morbidities (%)			
No co-morbidities	70.2 (1364/1943)	73.93 (5505/7446)	0.001
One co-morbidity	19.15 (372/1943)	17.94 (1336/7446)	0.222
Two co-morbidities	7.57 (147/1943)	6.24 (465/7446)	0.039
Three or more co-morbidities	3.09 (60/1943)	1.88 (140/7446)	0.002
Chronic cardiovascular disease	9.11 (177/1943)	5.77 (430/7446)	<0.001
Chronic hematologic disease	1.44 (28/1943)	0.79 (59/7446)	0.011
Chronic liver disease	0.62 (12/1943)	0.20 (15/7446)	0.006
Chronic neurological disease	0.72 (14/1943)	0.62 (46/7446)	0.631
Chronic pulmonary disease	0.98 (19/1943)	0.69 (51/7446)	0.183
Chronic renal disease	0.88 (17/1943)	0.48 (36/7446)	0.059
Asthma	3.76 (73/1943)	3.79 (282/7446)	>0.999
Cancer	0.31 (6/1943)	0.23 (17/7446)	0.605
Diabetes	7.36 (143/1943)	7.47 (556/7446)	0.923
Down syndrome	0.41 (8/1943)	0.05 (4/7446)	0.001
Endocrine disease	1.24 (24/1943)	1.69 (126/7446)	0.186
Gestational diabetes	1.39 (27/1943)	1.65 (123/7446)	0.477
HIV	0.26 (5/1943)	0.12 (9/7446)	0.184
Immunocompromised	1.49 (29/1943)	0.97 (72/7446)	0.049
Maternal hypertensive disorder	5.4 (105/1943)	4.15 (309/7446)	0.018
Mental disorder	0.21 (4/1943)	0.34 (25/7446)	0.492
Obesity	9.37 (182/1943)	7.48 (557/7446)	0.007
Respiratory viral infection	0.15 (3/1943)	0.19 (14/7446)	>0.999
Healthcare-related (%)			
ICU admission	38.70 (752/1943)	28.62 (2131/7446)	<0.001
Ventilation	53.78 (1045/1943)	48.23 (3591/7446)	<0.001
Antiviral	9.73 (189/1943)	11.24 (837/7446)	0.060
Vaccination against influenza	14.41 (280/1943)	15.79 (1176/7446)	0.139
Vaccination against SARS-CoV-2	4.12 (80/1943)	5.14 (383/7446)	0.068
Metropolitan region	59.86 (1163/1943)	60.88 (4533/7446)	0.419
Obstetric centre in establishment	81.01 (1574/1943)	86.31 (6427/7446)	<0.001
Private healthcare (for-profit)	16.46 (318/1932)	18.49 (1367/7395)	0.040
Private healthcare (not-for-profit)	30.18 (583/1932)	31.71 (2345/7395)	0.205
Public healthcare	53.36 (1031/1932)	49.80 (3683/7395)	0.005
UPA admission	3.1 (32/1031)	3.8 (140/3683)	0.347

Significant difference was also observed in some epidemiologic measures. These included shorter time from symptom onset to admission (5 vs 6* *days, *P *<* *0.001) and shorter time from admission to recovery (5 vs 6* *days, *P *=* *0.001) in the post-partum group; and a higher proportion of nosocomial infection (2.8% vs 0.9%, *P *<* *0.001) in the post-partum group.

The symptom profile also significantly differed. In particular, signs and symptoms that usually indicate a severe clinical course were more prominent in the post-partum group, such as abnormal chest X-ray (93.0% vs 86.6%, *P *<* *0.001) and low oxygen saturation (45.1% vs 38.4%, *P *<* *0.001). However, dyspnea was more prevalent in pregnant patients (56.2% vs 59.8%, *P *=* *0.003). Symptoms revealing upper respiratory tract infection were less prominent in post-partum patients including coryza (7.6% vs 9.5%, *P *=* *0.008), cough (62.7% vs 72.7%, *P *<* *0.001) and sore throat (18.7% vs 21.3%, *P *=* *0.016).

The post-partum group also had a higher prevalence of pre-existing health conditions. About 70% of the patients in the post-partum group had no co-morbidities compared with 75% in the pregnant group (*P *=* *0.001). Furthermore, the post-partum group had a higher share of individuals with two co-morbidities (7.6% vs 6.2%, *P *=* *0.039) and three or more co-morbidities (3.1% vs 1.9%, *P *=* *0.002). Several co-morbidities were more prevalent in the post-partum group. For instance, patients in post-partum had a 1.6-, 1.8- and 3.1-fold higher prevalence of chronic cardiovascular (9.1% vs 5.8%, *P *<* *0.001), hematologic (1.4% vs 0.8%, *P *=* *0.011) and liver (0.6% vs 0.2%, *P *=* *0.006) diseases. Other pre-existing conditions such as hypertensive disorder (5.4% vs 4.2%, *P *=* *0.018) and obesity (9.4% vs 7.5%, *P *=* *0.007) were also found to be more prevalent in the post-partum group.

For healthcare-related measures, patients in post-partum were 1.4 and 1.1 times more likely to be admitted to the ICU (38.7% vs 28.6%, *P *<* *0.001) and required mechanical ventilation (53.8% vs 48.2%, *P *<* *0.001), respectively. There were also differences in the type of healthcare providers between the two groups. Patients in post-partum were less likely to be hospitalized by private healthcare providers (16.5% vs 18.5%, *P *=* *0.040) and less likely to be admitted to hospitals that had an obstetric centre (81.1% vs 86.3%, *P *<* *0.001). For those who were hospitalized in the public healthcare sector, 3.8% and 3.1% of patients in pregnancy and post-partum were admitted to the UPA, respectively, yet the difference was not significant (*P *=* *0.347).

**Figure 2:**
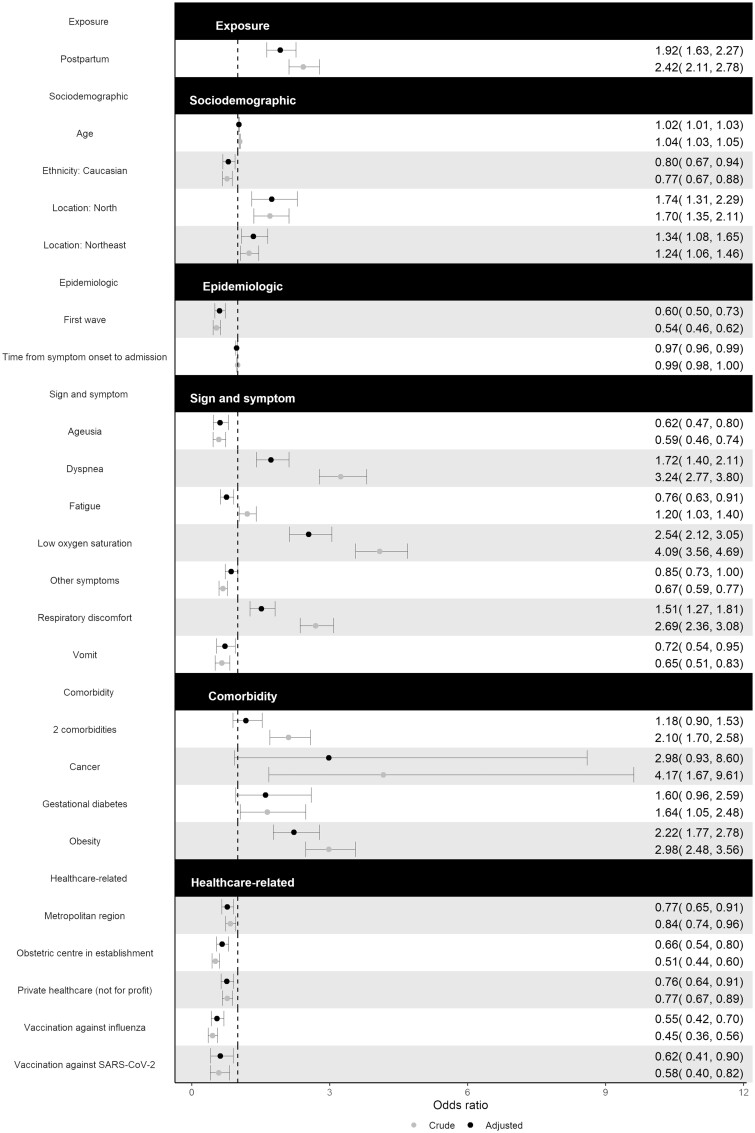
Results of multivariable logistic regression model

Results of the multivariable logistic regression are shown in Figure 2. Patients in post-partum had almost twice the odds (adjusted OR 1.92, 95% CI 1.63–2.27) of succumbing to SARS-CoV-2 infection even after adjusting for socio-demographic, epidemiologic, clinical and healthcare-related factors. Other factors that were identified as predictors of COVID-19-related in-hospital death included dyspnea (adjusted OR 1.72, 95% CI 1.40–2.11), low oxygen saturation (adjusted OR 2.54, 95% CI 2.12–3.05) and respiratory discomfort (adjusted OR 1.51, 95% CI 1.27–1.81). For socio-demographic factors, Caucasian patients, as an ethnic minority, had a 20% decrease in the odds of death (adjusted OR 0.80, 95% CI 0.67–0.94) compared with Hispanic/African patients. Patients in the North and Northeast Regions had a 74% (adjusted OR 1.74, 95% CI 1.31–2.29) and 34% (adjusted OR 1.34, 95% CI 1.08–1.65) increase in the odds of death, respectively.

Several healthcare-related factors were predictors of in-hospital mortality. Patients admitted to a hospital located in a Metropolitan Region had a 23% (adjusted OR 0.77, 95% CI 0.65–0.91) decrease in the odds of death compared with those admitted to a hospital located in a rural area. Furthermore, patients admitted to a hospital operated by a private not-for-profit healthcare provider had a 24% (adjusted OR 0.76, 95% CI 0.64–0.91) decrease in the odds of death compared with one operated by the SUS. Patients admitted to a hospital that had an obstetric centre had a 34% (adjusted OR 0.66, 95% CI 0.54–0.80) decrease in the odds of death compared with one that did not.

Results of the sensitivity analysis are shown in Supplementary Figure S1 (available as Supplementary data at *IJE* online). The ORs calculated based on severe cases are similar to those based on all cases, as demonstrated by the 95% CIs. This implies that the effect of a lower threshold for hospitalization of pregnant women was very small. The area under the ROC curve is reported to be 0.775 (95% CI 0.761–0.788), indicating good accuracy. VIFs are reported in Supplementary Table S1 (available as Supplementary data at *IJE* online) and no signs of serious collinearity were detected.

**Figure 3:**
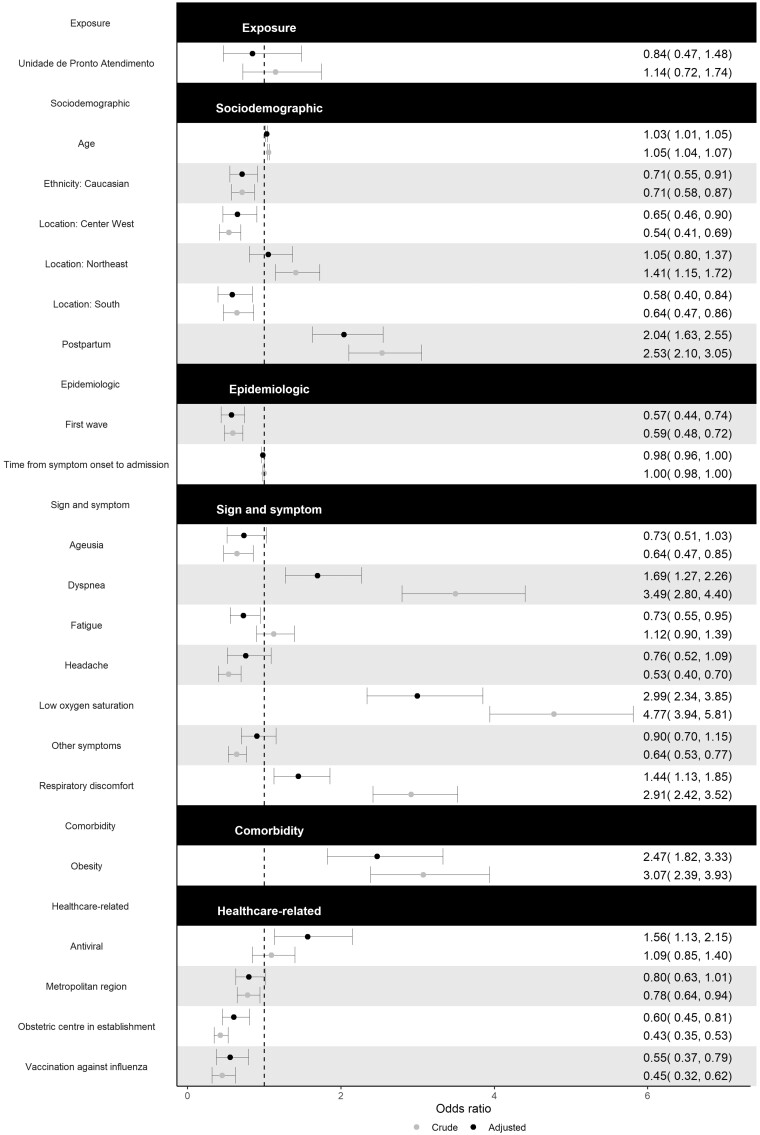
Results of multivariable logistic regression model (only those admitted to public hospitals)

Results of the multivariable logistic regression confined to patients admitted to public hospitals are shown in Figure 3. Patients admitted to the UPA did not have significantly higher odds of COVID-19 in-hospital mortality (adjusted OR 0.84, 95% CI 0.47–1.48). Again, patients in post-partum had about twice the odds (adjusted OR 2.04, 95% CI 1.63–2.55) of succumbing to SARS-CoV-2 infection even after adjusting for socio-demographic, epidemiologic, clinical and healthcare-related factors. Furthermore, patients admitted to a public hospital that had an obstetric centre had a 40% (adjusted OR 0.60, 95% CI 0.45–0.81) decrease in the odds of death compared with those that did not. Results of the sensitivity analysis are shown in Supplementary Figure S2 (available as Supplementary data at *IJE* online). The ORs calculated based on severe cases are similar to those based on all cases, as demonstrated by the overlapping 95% CIs. The area under the ROC curve is reported to be 0.793 (95% CI 0.775–0.810), indicating good accuracy. VIFs are reported in Supplementary Table S2 (available as Supplementary data at *IJE* online) and are <5, suggestive of the lack of serious collinearity.

## Discussion

Based on the data of >9000 pregnant or post-partum COVID-19 hospitalized patients gathered from the national registry in Brazil, we demonstrated that the post-partum period was associated with an increased risk of COVID-19-related in-hospital mortality. Furthermore, there was no significant difference in the in-hospital mortality risk between patients admitted to UPAs and other establishments within public healthcare.

Our findings are in line with existing literature. A number of case series have highlighted COVID-19-related post-partum exacerbations. Reported in An *et al.*,^22^ all three pregnant women with SARS-CoV-2 developed post-partum dyspnea (including one who already had dyspnea during pregnancy) and had a further decrease in oxygen saturation in post-partum. The authors suggested that the exacerbation might be associated with changes in chest imaging. A case series in Turkey reported four patients with oxygen saturation decreased and D-dimer increased who died during the post-partum period. The author noticed that all four patients delivered via caesarean section and suggested that caesarean section might be a surgical burden that led to exacerbation.^23^ Similarly, two of the three patients reported in An *et al.* delivered via caesarean section. However, caesarean section per se might not be associated with the exacerbation. In the above-mentioned cases, caesarean section was conducted upon the advice of obstetricians based on a number of considerations such as maternal health. Therefore, patients might already be in a severe state and the decision for caesarean section might merely indicate exacerbations.

Unfortunately, data of the mode of delivery were not available in the database. Thus, we cannot examine whether the increased risk of COVID-19 mortality in the post-partum group was genuinely due to caesarean section. Brazil has one of the highest rates of caesarean section in the world. About 56% of the live births in the country between 2014 and 2017 were via caesarean section^24^ and it was suggested to be even higher during the pandemic.^25^ Nevertheless, mothers who delivered by caesarean section were more likely to come from urban areas, to be Caucasian and to have attended private clinics^26^^,^^27^—factors that were rather shown to be associated with lower mortality risk.

Certain co-morbidities such as diabetes that have been suggested to be associated with increased risk of COVID-19 death in existing literature^28^ were not identified as predictors of death in the present model. This might be attributed to the choice of confounders, including healthcare-related variables in which certain healthcare might be effective in reducing the risk of COVID-19 death associated with diabetes.

Nevertheless, this finding can also suggest that post-partum COVID-19 deaths are preventable through quality healthcare. Adjusted for age, ethnicity, co-morbidities and the use of intensive care, an earlier study on pregnant and post-partum women in Brazil dated up to 18 June 2020 found that post-partum women had 2.5 higher odds of succumbing to SARS-CoV-2 infection^28^—higher than the OR of 1.9 reported in the present work. The discrepancy might be attributed to the inclusion of healthcare-related covariates in our work. If better-quality healthcare can reduce post-partum mortality, it may explain why post-partum COVID-19 deaths have only been observed in limited countries—an example of the inverse data law in which those most at risk are least likely to be counted.^29^

Additionally, UPAs appeared to provide care that was as effective as other healthcare facilities, as shown in our analysis. They have also been shown to reduce geographical inequality in access to healthcare within metropolitan areas.^30^ However, the significance of the location variables such as the Northeast, North and Metropolitan Regions indicated that the accessibility problem persists in the northern states and rural areas. The lack of hospital beds remains a problem in Brazil despite efforts to increase the number of beds during the pandemic. Given the effectiveness in reducing COVID-19 mortality, UPAs should be given a key role in the fight against the pandemic. For example, temporary UPAs can be set up in rural areas to help in triaging patients, preventing unnecessary travel for patients who do not require hospital care.

Although the present work did not investigate the biological mechanism behind the increased risk of mortality in puerperal patients, we speculated that the finding might be attributed to conditions that occur more often in post-partum than in pregnancy, such as venous thromboembolism (VTE). VTE can occur a any time during pregnancy but post-partum is the time of highest risk, with a relative risk of ∼20-fold.^31^ Moreover, post-partum infection intensifies the risk of VTE. It was found that patients with post-partum infection hospitalized for pregnancy-related reasons were seven times more likely to be diagnosed with VTE compared with those without.^32^ For SARS-CoV-2 infection, a systematic review found that thromboembolism (predominately VTE^33^) is a risk factor for COVID-19-related mortality in the general population (OR 1.74, 95% CI 1.01–2.98).^34^ The interplay between infection and VTE remains an active research area. So far, it has been suggested that the immune and coagulation systems interact and reciprocally regulate one another.^35^ Nevertheless, the diagnosis of VTE in pregnant and post-partum women remains challenging. There are still no reliable and consensual reference ranges for D-dimer for this population group due to the elevation and varying of D-dimer during pregnancy and post-partum.^36^

The present work has some limitations. As discussed, data on the mode of delivery were not available, limiting further investigation on the impact of caesarean section. Furthermore, detailed measures were not available given that the database was for disease surveillance. Some measures, including laboratory parameters, are useful in ascertaining the underlying factors of mortality in post-partum patients. For instance, D-dimer together with ultrasonography findings can provide a clinical picture of VTE. Finally, missing data was another issue, commonly seen in registry data. For instance, patients being hospitalized might have the clinical status considered as ‘not available’ and therefore were excluded for analyses. However, we believe that in our analyses the main reason for the missing data in clinical endpoints was administrative due to the study cut-off date. Therefore, the assumption of ‘missing completely at random’ is plausible and the study results are valid for the study populations. In addition, under-reporting is another limitation. Universal testing for SARS-CoV-2 infection was not available for the obstetric population until August 2020 and was only offered to symptomatic pregnant patients with access to healthcare, leading to case under-reporting in poor communities.

To conclude, it was shown that puerperium was associated with an increased risk of COVID-19-related in-hospital mortality. Nevertheless, certain types of healthcare were shown to be associated with lower mortality risk. Therefore, to reduce the risk of COVID-19 in-hospital post-partum maternal death, healthcare that was offered in obstetric centres and to obstetric patients in urban areas should be made more accessible. Furthermore, a number of unanswered questions warrant further investigation, including the underlying biological mechanism of post-partum of COVID-19 deaths, such as venous thromboembolism, as well as the impact of the mode of delivery on post-partum SARS-CoV-2 infection.

## Ethics approval

Ethics approval was not required in Brazil and UK because the data were de-identified and publicly available.

## Supplementary Material

dyac157_Supplementary_Data

## Data Availability

The data underlying this article will be shared on reasonable request to the corresponding author.
