# Supplementary material for: Better healthcare can reduce the risk of COVID-19 in-hospital post-partum maternal death: evidence from Brazil
Source: Int J Epidemiol. 2022 Aug 10;51(6):1733–44. doi: 10.1093/ije/dyac157 (PMC9384644; doi:10.1093/ije/dyac157)

Supplementary materials

Supplement to: Leung C, Simões e Silva AC, Su L. Better healthcare can reduce the risk of COVID-19 in-hospital postpartum maternal death: Evidence from Brazil.

Table S1. Estimated variance inflation factors (primary outcome)

	VIF
Postpartum	1.04
Age	1.03
Ethnicity: Caucasian	1.26
Location: North	1.19
Location: Northeast	1.23
First wave	1.14
Time from symptom onset to admission	1.06
Ageusia	1.04
Dyspnoea	1.28
Fatigue	1.16
Low oxygen saturation	1.34
Respiratory discomfort	1.31

	VIF
Vomit	1.02
Other symptoms	1.05
2 comorbidities	1.18
Cancer	1.02
Obesity	1.16
Gestational diabetes	1.03
Vaccination against influenza	1.04
Vaccination against SARS-CoV-2	1.02
Private healthcare	1.19
Metropolitan region	1.20
Obstetric centre in establishment	1.10

Note: VIF stands for variance inflation factors

Table S2. Estimated variance inflation factors (secondary outcome)

	VIF
Postpartum	1.06
Age	1.03
Ethnicity: Caucasian	1.24
Location: Northeast	1.24
Location: Center West	1.21
Location: South	1.28
First wave	1.28
Time from symptom onset to admission	1.08
Ageusia	1.05
Dyspnoea	1.26
Fatigue	1.20

	VIF
Headache	1.32
Low oxygen saturation	1.31
Respiratory discomfort	1.32
Other symptoms	1.37
Obesity	1.07
Antiviral	1.23
Vaccination against influenza	1.03
Metropolitan region	1.18
Obstetric centre in establishment	1.47
Unidade de Pronto Atendimento	1.43

Note: VIF stands for variance inflation factors

Figure S1. Sensitivity analysis for the primary outcome

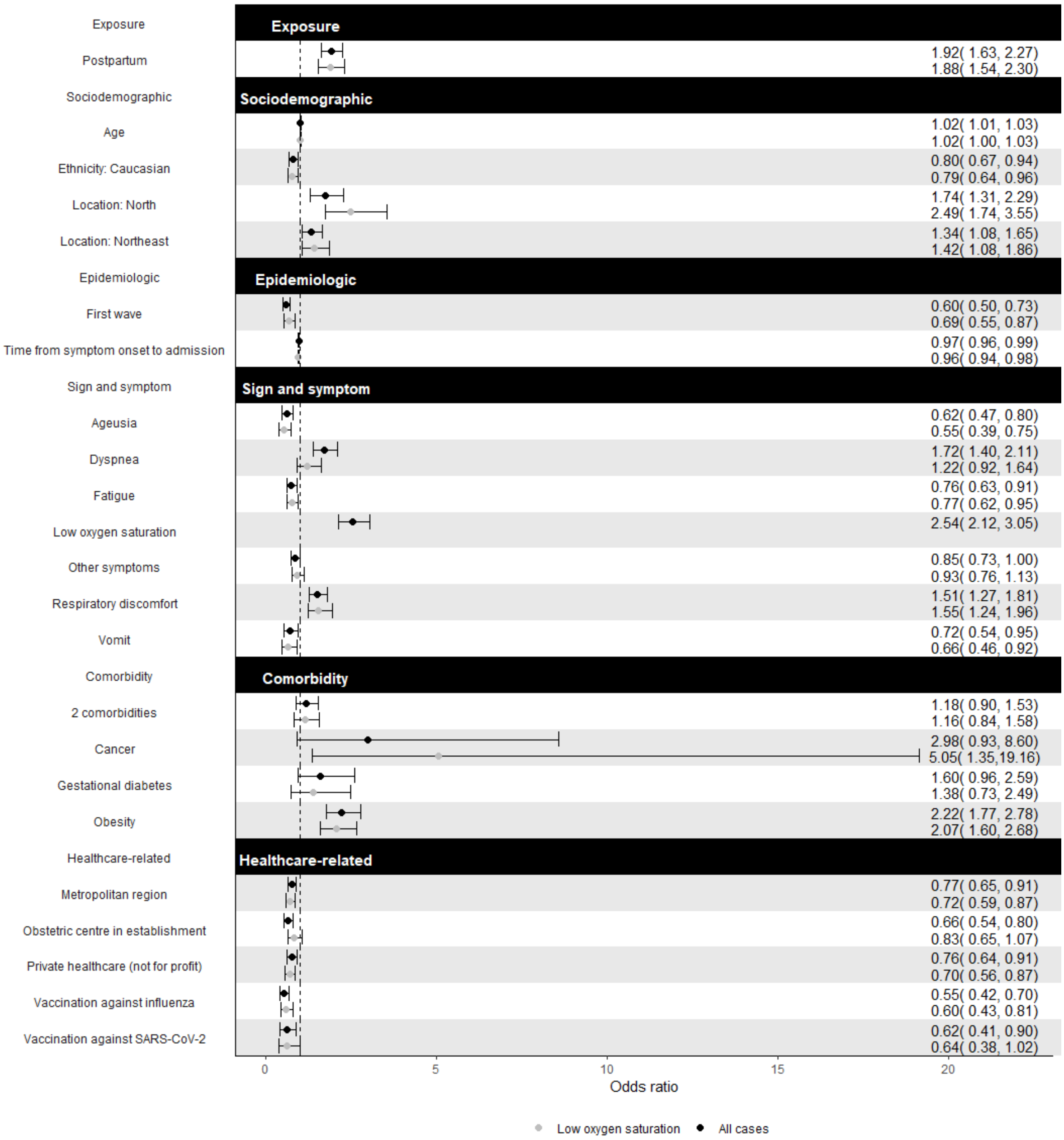

Figure S2. Sensitivity analysis for the secondary outcome

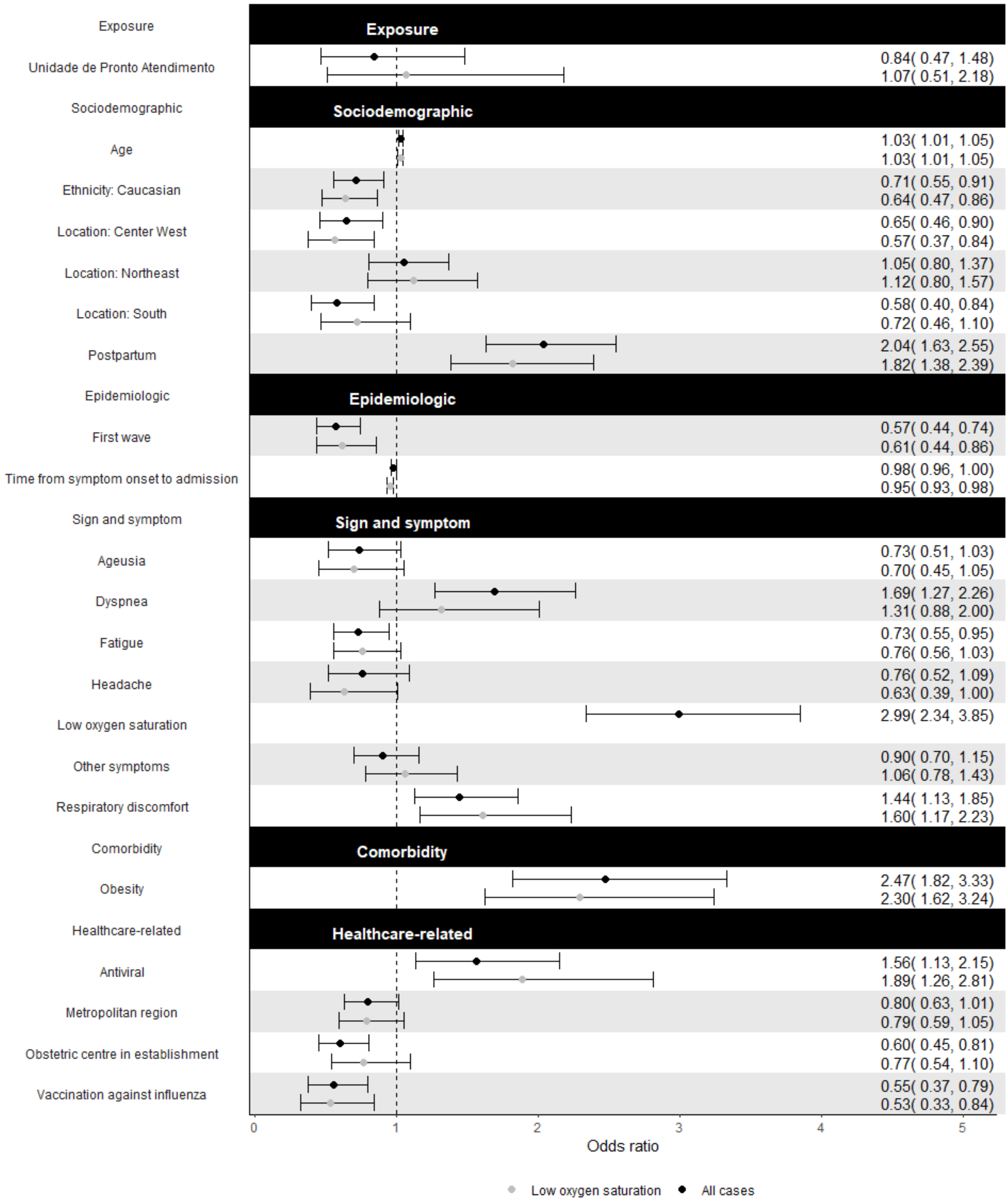

Supplement: dyac157_Supplementary_Data [file dyac157_supplementary_data.pdf]
